# MagicFinger: 3D Magnetic Fingerprints for Indoor Location

**DOI:** 10.3390/s150717168

**Published:** 2015-07-15

**Authors:** Daniel Carrillo, Victoria Moreno, Benito Úbeda, Antonio F. Skarmeta

**Affiliations:** Department of Information and Communications Engineering, University of Murcia, 30100 Murcia, Spain; E-Mails: mvmoreno@um.es (V.M.); bubeda@um.es (B.Ú.); skarmeta@um.es (A.F.S.)

**Keywords:** indoor location, smartphone, magnetic field, fingerprinting

## Abstract

Given the indispensable role of mobile phones in everyday life, phone-centric sensing systems are ideal candidates for ubiquitous observation purposes. This paper presents a novel approach for mobile phone-centric observation applied to indoor location. The approach involves a location fingerprinting methodology that takes advantage of the presence of magnetic field anomalies inside buildings. Unlike existing work on the subject, which uses the intensity of magnetic field for fingerprinting, our approach uses all three components of the measured magnetic field vectors to improve accuracy. By using adequate soft computing techniques, it is possible to adequately balance the constraints of common solutions. The resulting system does not rely on any infrastructure devices and therefore is easy to manage and deploy. The proposed system consists of two phases: the offline phase and the online phase. In the offline phase, magnetic field measurements are taken throughout the building, and 3D maps are generated. Then, during the online phase, the user's location is estimated through the best estimator for each zone of the building. Experimental evaluations carried out in two different buildings confirm the satisfactory performance of indoor location based on magnetic field vectors. These evaluations provided an error of (11.34 m, 4.78 m) in the (*x, y*) components of the estimated positions in the first building where the experiments were carried out, with a standard deviation of (3.41 m, 4.68 m); and in the second building, an error of (4 m, 2.98 m) with a deviation of (2.64 m, 2.33 m).

## 1. Introduction

In indoor environments, such as buildings, obtaining precise location information is still a challenging task. Traditional mechanisms, such as GPS [1], are unworkable inside buildings, due to the attenuation of the satellite signal. This has resulted in the development of alternative indoor location systems with acceptable results, such as those based on WiFi [2], ZigBee [3] and RFID [4]. Nevertheless, a common requirement of these approaches is the need for specific devices to be deployed or additional hardware to be used during the location process. Consequently, these solutions are expensive, and regular maintenance is required. Furthermore, the limited accuracy of the indoor location solutions proposed to date is another issue that needs to be addressed. For example, when wireless technologies are used to solve indoor location problems, physical obstacles produce signal interferences that affect the performance of the location system in question.

As an alternative, the potential use of exploiting mobile phones for sensing and context recognition has recently attracted interest from researchers in both the industrial and academic communities [5]. This has resulted in a huge range of new solutions for indoor location-related problems [6]. The ubiquitous and longitudinal data that smartphones can provide are expected to revolutionize technological services and spark a new wave of ubiquitous services.

Modern mobile phones contain a number of sensors capable of sensing the user's location and the presence of nearby entities. Apart from GPS, which is primarily used for outdoor positioning, GSM and Wi-Fi signals or iBeacons based on Bluetooth Low Energy (BLE) technology can also be used for user location (for an extensive overview of ubiquitous location, refer to [7]). In addition, integrated sensors in smartphones, such as inertial and ambient sensors, are also being exploited to solve location problems [8].

In this work, we present MagicFinger: a novel approach to indoor location using the magnetometers that are integrated in common smartphones. Unlike most infrastructure-based solutions [7], our system does not rely upon any additional support infrastructure. Our solution only requires a personal smartphone able to sense the magnetic field present inside buildings, *i.e.*, a phone that includes a magnetometer in its set of sensors. However, using nothing more than a mobile phone to predict user location restricts the accuracy of the proposed approach. For instance, it is difficult to estimate the user's location in areas inside a building that show low variability in their magnetic field distribution, as well as in zones whose magnetic field values fluctuate excessively. Despite these inconveniences, our proposed solution can cover most of the space within a building, resulting in a cost-effective, scalable system.

The methodology consists of two phases. First, maps containing the magnetic field profile of the building in which the location problem is to be solved are generated; this constitutes the offline training phase of the system. Then, during the online phase, users supply the system with the magnetic field vectors measured by their phones, and based on these measurements, the system is able to provide accurate location data.

The structure of this paper is as follows: Section 2 describes the foundations that support the feasibility of using magnetic field measurements from smartphones to solve indoor location problems and presents related works. Section 3 analyzes the main drawbacks of these proposals and presents our solution. In Section 4, the goals of this work and the methodology followed to develop the system are formalized. This section explains the stages of the indoor location system, which is based on an offline phase to generate the location model, followed by an online phase where position is estimated in real time. Section 5 details the scenario where we carry out the tests to evaluate the system, the experimental results obtained and the validation of the system in a different building. Finally, Section 6 provides conclusions and a description of potential future directions of our work.

## 2. Related Work

Certain elements inside buildings can distort or even drown out the weak direction of the Earth's magnetic field, which is used for navigation and location purposes [9]. The effect of these elements can be divided into deterministic interference, which includes the effect of ferrous materials (soft iron) and magnetized materials (hard iron), and non-deterministic interference.

Location proposals based on magnetic field measurements assume that the success in estimating orientation and position is conditioned by the capacity of magnetometers to sense the Earth's magnetic field in environments containing magnetic anomalies. In principle, a non-uniform indoor ambient magnetic field produces different magnetic observations depending on the path taken through it. In other words, static objects or infrastructures inside buildings (such as steel structures, electric power systems and electronic and mechanical appliances) perturb the Earth's magnetic field and can make up a profile of magnetic field values (a map composed of magnetic field fingerprints), which can be used to solve the location problem.

Previous studies on indoor location systems using magnetic field measurements, e.g., [10], identified landmarks of increased magnetic intensity resulting from common indoor objects, such as large pillars or vending machines. In the same work, magnetic field intensity datasets taken in the corridors at the University of North Texas were analyzed, and the magnetic signatures identified were found to be stable over time. The study used a variety of phones at different sensitivity rates, all of which provided similar results. They concluded that the magnetic signatures used for identifying zones and rooms in buildings are unaffected by the people, phones and the sensor sensitivity rates involved. Furthermore, in [11], the feasibility of using the magnetic field alone for indoor location was fully studied. The authors first validated the stability of the indoor geomagnetic field over a long period of time and concluded that the intensity of the Earth's magnetic field is not the same in different zones of a given building, mainly because of the presence of steel structural elements, ferromagnetic objects and the electronic devices typically found there. These elements perturb the Earth's magnetic field significantly, which provides an advantage for distinguishing different locations inside buildings. Furthermore, potential interferences were investigated. For this, the authors selected several objects and assessed how the magnetic field perturbation decreased very rapidly as the distance from the interfering source increased. From these tests, they demonstrated that the size of the object responsible for the interference had a direct impact on the perturbation generated (the larger the object, the greater the distance needed for the perturbation to decrease). The authors concluded that using the magnetic field for indoor location purposes in buildings containing a few elements that are useful for generating magnetic field profiles may result in low accuracy in the location data, as would be the case in buildings with a low level of electronic or ferrous infrastructures. To mitigate this drawback, Angermann, M. *et al.* [12] analyzed the resulting omnipresent and rich three-dimensional information of magnetic fields, which can support higher-dimensional location estimates. They showed how an indoor magnetic field exhibits a fine-grained and persistent micro-structure of perturbation in terms of direction and intensity. The vectorial properties of the magnetic field inside buildings were analyzed, considering these to be time-invariant and variable in direction and intensity over the three-dimensional free space surrounded by floors, walls or physical objects, such as furniture or machinery. Nevertheless, it is important to note that the authors made experiments in very restricted areas of a building, and they themselves encouraged investigation over wider areas. The study concluded by emphasizing the importance of addressing the resolution of magnetic maps, sensor noise and possible temporal changes in the magnetic field in future works.

Chung, J. *et al.* [13] analyzed the feasibility of using a four-magnetometer system attached to the chest of a person for locating purposes without the need to predict the orientation of the person. For this to be accomplished, they collected test data by rotating a chair, with their sensors affixed to it, at every measured location, which enabled them to obtain fingerprinting information in a variety of directions. Furthermore, their work took advantage of the three components of magnetic fields, confirming that this is a more flexible proposal than when only the intensity of magnetic field is used. For the latest research developments on indoor location through magnetism, see Chapter 4 of [14].

Based on all of these studies, it can be affirmed that indoor magnetic profiles need to be well characterized and quantified prior to any estimation process [15]. As mentioned above, to date, most efforts have been directed at studying the feasibility of using the indoor magnetic field for location purposes, concluding that it represents a stable and unique solution applicable to this problem as long as the infrastructure of the building remains stable. However, very few indoor location systems following this approach have been proposed and evaluated [11,12]. Furthermore, the scalability and robustness of location solutions based on this approach in buildings are open issues that require to be further addressed, especially in buildings where the number of magnetic field perturbation sources is low or when these sources are of the same nature. Another important aspect to consider is that in some countries (as is common in Europe), there is a strong governmental control concerning the maximum magnetic field values that electronic and mechanical devices can generate [16]. Bearing all these aspects in mind, in Section 4, we present the MagicFinger mechanism, which uses the three magnetic field components sensed by smartphone magnetometers to provide a robust and accurate indoor location system for buildings.

## 3. Preliminary Study

In this section, we start by analyzing the magnetic field profile of a building, namely the first floor of the Computer Science Faculty of the University of Murcia (Spain), considering the intensity value alone. The aim of this initial experiment is to try to show that the variation in the magnetic field intensity is not as relevant as one might expect, despite the number of magnetic field perturbation sources identified *a priori*, e.g., electronic machines that are installed in the environment.

**Figure 1:**
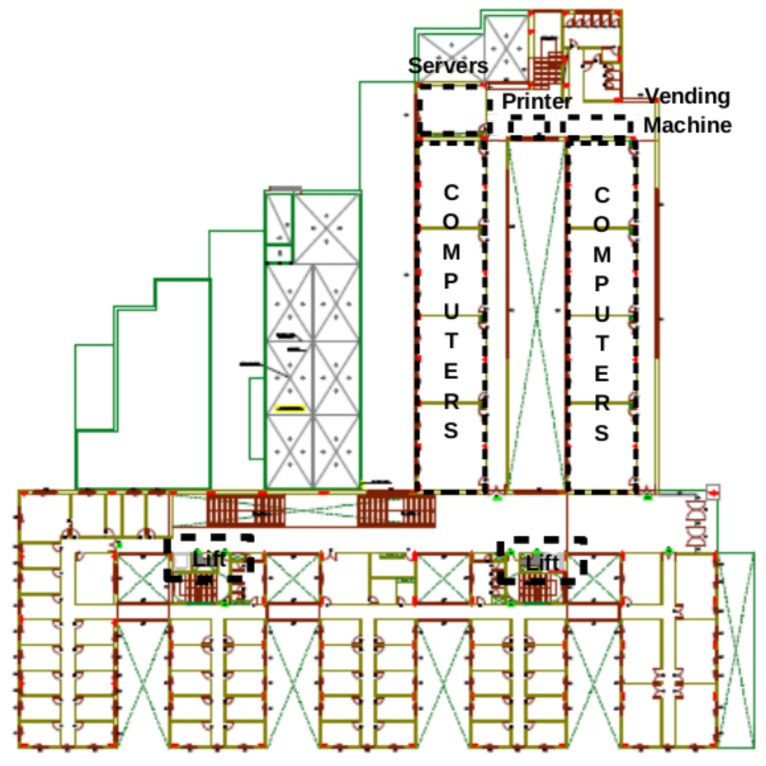
Floor where the preliminary study was carried out (Building A).

**Figure 2:**
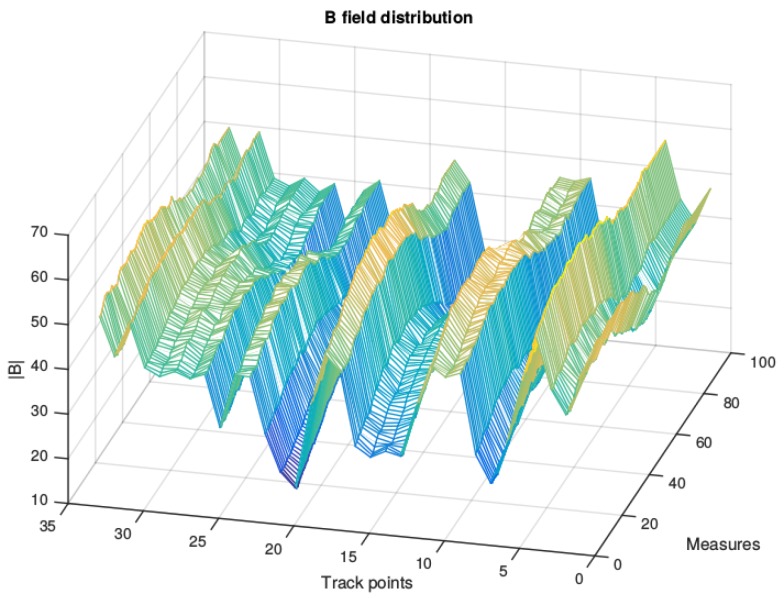
Distribution of the magnitude of the magnetic field along a corridor.

On this particular floor, several zones, such as a room with a large number of servers, computers, printers and lifts in different locations, are regarded as candidates for acting as magnetic field landmarks. Some of these locations are identified with dotted lines in Figure 1. The measurement system used to gather the data consists of an HTC One X phone equipped with a Hall-effect magnetometer, and the data acquisition frequency is 13 Hz. Samples are collected horizontally along a 50-m corridor by rotating a platform at each measurement point. The distance between each pair of measurement points is set to 150 cm. By analyzing the collected values for the magnetic field intensity (see Figure 2), a standard deviation of 7.6 μT and a difference of 55 μT between the maximum and the minimum of the magnetic field intensity is obtained. Therefore, we can assume that in a common building, such as a house, where the number of infrastructures installed would be lower, magnetic field variability alone would not be sufficient to achieve accurate position estimates.

**Figure 3:**
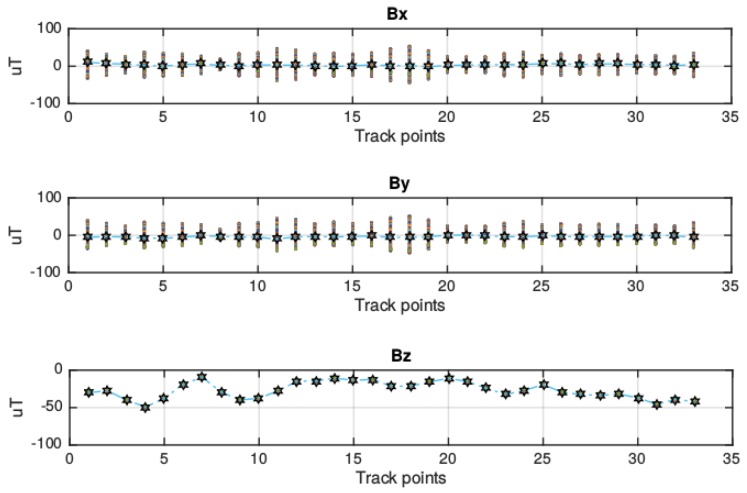
Magnetic field profiles of the corridor.

Because of this restriction, the variability existing in the values of the three components of the magnetic field is analyzed. Figure 3 shows the distribution of the magnetic field for each component. As can be seen, the mean of component *z* depends on the location in the corridor. Components *x* and *y* have a similar mean due to the rotation of the platform. This is because the coordinate system of the sensor is defined relative to the device's screen. Nevertheless, it is interesting that each measurement point has a different range of values for these two components. From these profiles, it can be seen that a more complete characterization of the building can be provided if each point is represented by the three components of the magnetic field sensed at that point. A similar conclusion was reached in [12], in which the authors showed how the magnetic field vector formed by the three components of the magnetic field measured during an experiment traced non-overlapping curves in the space, which means magnetic vectors can be considered sufficiently informative to prevent any ambiguity when location is being estimated.

This approach represents an alternative solution to the limitations of using only the magnitude. Nevertheless, it is important to note the limitation of our solution, as it requires the height of the mobile phone to be known and controlled during the measurement process. More specifically, the height of the phone must remain constant during the construction of the map and the online phase of the location estimation. This is necessary to avoid any ambiguity, since all values of the magnetic field components will be referenced to the same coordinate system. It could be argued that the orientation of the mobile phone also needs to remain constant; however, we are interested in measuring every orientation at a fixed height. The reason for this is that predicting the person's orientation is not as important as knowing their location inside the building, which is our main goal. Such an approach was proposed by Chung, J. *et al.* in [13].

Moreover, a relevant issue for any location mechanism is the data processing techniques that are able to learn the profile of each zone within the building. Effectively addressing this problem can minimize the error in estimating the user location and can make the mechanism adjustable to each particular building. Furthermore, in magnetic field-based location problems, any uncertainty in the estimation of location must be dealt with, because accuracy varies depending on the building zone where the user is located. Therefore, it is necessary to apply suitable soft computing techniques to cope with this difficulty in order to achieve accurate location estimates. For all of these reasons, the computation techniques chosen in this work are based on scene analysis, where reference measurements (fingerprints) of the ambient magnetic field are first collected and processed to generate 3D magnetic field maps of the building (offline training phase of the MagicFinger). Then, the user's location is estimated by matching online measurements with the closest *a priori* location fingerprints (online phase of the MagicFinger). These two phases are fully explained in the next section.

## 4. MagicFinger: An Indoor location Mechanism Based on 3D Magnetic Fingerprints

### 4.1. Methodology

As demanded in several surveys, it is necessary to test the feasibility of using magnetic fields alone for location purposes in different environments. The goals of our research can be summarized as follows: (i) to validate magnetic field-based location solutions in a real environment; (ii) to design a scalable solution that can be applied to a whole building without the need to deploy new infrastructure; and (iii) to study whether the proposed solution can be generalized to other types of buildings or should rather be particularized to a specific scenario. Then, this work will provide information on the applicability of magnetic fingerprinting in real-world conditions.

For (i) and (iii) to be accomplished, data will be collected in the target buildings, in our case the first floor of the Computer Science Faculty (Building A) and the ground floor of the Psychology Faculty (Building B), of the University of Murcia. These scenarios have different building infrastructures and activity levels, so the performance of our system can be assessed in two environments with dissimilar conditions.

Regarding the design of a non-infrastructure-based location solution, our aim is to generate models of 3D magnetic field patterns in buildings. To build these models, several different techniques are available. We decided to generate the 3D magnetic field maps by soft computing techniques for accurate and reliable location estimates, as they are widely used by the research community. A general description of the different actions to be performed to generate such location models for buildings is presented below.

1. Data collection: Magnetic field data are gathered using a smartphone with a built-in magnetometer. It is important to take into account that such magnetometer readouts are prone to disorientation of the sensing module and are dependent on orientation and position of the user's phone during the data collection process, e.g., the device may be in a trouser pocket or in a bag. Thus, it is necessary that these aspects are correctly associated with the data collection performed, which should be considered in the offline phase of the MagicFinger mechanism. Note that if user orientation is to be predicted, users' orientation should also be associated with data.
2. Clustering: The data gathered are processed to identify zones of the building where the magnetic field distribution presents peaks and similarities that can be used as magnetic field landmarks. These landmarks will be used later to geo-reference the magnetic field values measured in the building during the online phase of MagicFinger.
3. Classification: A classifier learns the space division according to the landmarks identified in the previous step. The partitioning of the space into zones makes the estimation much more accurate and also makes the system scalable.
4. Estimation: Each of these zones is analyzed in terms of magnetic field distribution associated with space distribution, and the best regression technique to estimate the user's location is implemented for each one of the zones of the building associated with a landmark.

**Figure 4:**
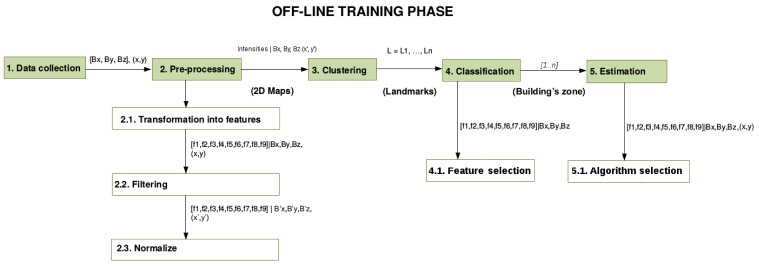
Offline phase of the MagicFinger mechanism.

Figure 4 presents a schema of the sequence of all of the above-mentioned steps. This represents the offline training phase of MagicFinger. The online phase corresponds to real-time predictions made by our system, and will be used to assess the accuracy of our proposal.

Several computational techniques recommended for solving each of these objectives are reviewed and considered. The techniques finally selected are described in the next section along with a description of the associated problem.

### 4.2. Offline Training Phase

#### 4.2.1. Data Collection

Due to the vectorial nature of the magnetic field, not only does the user's location inside the building have particular magnetic field vectors associated with it, but also the height at which the mobile phone is carried. The reason for this is that the magnetic field, and each of its components, depends inversely on the cubed distance from the source that is causing the magnetic field perturbation. This is why during this first stage, the carrying position of the user's phone is pre-established, to be later considered and associated with the appropriate magnetic field maps generated at the end of the offline training phase of the system. Thus, it is possible to generate descriptive models based on this information to identify the user's location.

Firstly, data were gathered at different heights throughout the building. During model training, the accuracy of all of these models was seen to be very similar, so different heights did not seriously affect the estimation of the user's location. Even though the magnetic field vectors depend on the distance from the magnetic sources, the distance between the possible places of the human body where a phone is carried do not differ much. Taking this into account, a height of 1.03 m is taken as the mean height at which people usually carry their phone. Then, “snapshots“ of the magnetic field are collected over short periods of time (less than a minute at each location *Z_q_*) and throughout the building. These snapshots are taken by rotating the sensor, gathering data every three or four degrees of rotation at a particular location *Z_q_*. Such measurements are then associated with the physical locations $Z_{q}^{\left(t\right)}$ where they were gathered. Several data collection sets are carried out, considering different context conditions, such as different levels of occupancy, different times of the day, *etc.*, so that the building models generated are sufficiently representative to cover different contextual conditions of the building. The data pairs are as follows:

(1) $$\left(B_{q}^{\left(t\right)},Z_{q}^{\left(t\right)}\right),q=1,2,\ldots ,N$$

where *N* is the number of data instances at location $Z_{q}^{\left(t\right)}\in {\mathbb{R}}^{2}$ and $B_{q}^{\left(t\right)}=\left[Bx_{q}^{\left(t\right)},By_{q}^{\left(t\right)},Bz_{q}^{\left(t\right)}\right]\in {\mathbb{R}}^{3}$ makes reference to the magnetic field vector in such location at instant *t*.

#### 4.2.2. Pre-Processing

The pre-processing unit is responsible for preparing the measured data by transformation. Moreover, feature vectors are extracted from the data for use in location estimation. The different processing techniques applied at this stage are as follows in Table 1:

1. Transformation: This is based on the raw dataset collected by the device sensor. During the transformation, compact representations of the magnetic field values, named features, are extracted. These are used later for location estimation. The values within the dataset are arranged into windows of between 80 and 100 samples, grouping the values at *Z_q_* which corresponds to the different orientations at that particular location. Each window is processed by several feature-extraction techniques, producing a feature vector that can be used to train the classifier and to feed the estimators. This selection of features representing magnetic field distributions is based on studies proposed in the literature that already use them for a similar purpose. At this stage, 27 features are extracted for evaluation (nine features for each magnetic field component: *Bx, By* and *Bz*).
2. Filtering: During this process, a filter is applied to remove features extracted from the training dataset that vary very little or too much.
3. Normalization: All values in the dataset are normalized. The resulting values are in the [0,1] range for every feature extracted form the initial dataset.
4. Feature selection: Besides considering only the intensity of the three components of the magnetic field for comparison purposes (since most works are based on this), we train the different classification and regression models with the most representative features for each model. For this, the features that have been filtered are ranked through recursive feature elimination by means of a resampling algorithm, as explained in [28]. This algorithm works by recursively removing predictors (in this case, features) from an initial set containing all of them and assessing the performance of every fitted model with the set of predictors considered at each iteration. In this way, it identifies the best set of features. The model used for measuring performance is random forests [29], since this technique is commonly used for feature selection. Finally, different features are used for clustering and for training the classifier and the estimators, particularly the features that prove to be the most appropriate for each technique.

#### 4.2.3. Clustering

During this stage, the space inside the building is divided according to the distribution of the magnetic field values, and the data collected are grouped according to the clusters identified. Note that for the proposed methodology to be independent of the building and, thus, scalable, data must be gathered throughout the building. By using the clustering step, the zones containing strong sources of perturbation (the zones expected to be landmarks) are identified correctly. If data were only gathered in these zones and not throughout the building, it would not be possible to ensure that all possible landmarks are identified.

**Figure 5:**
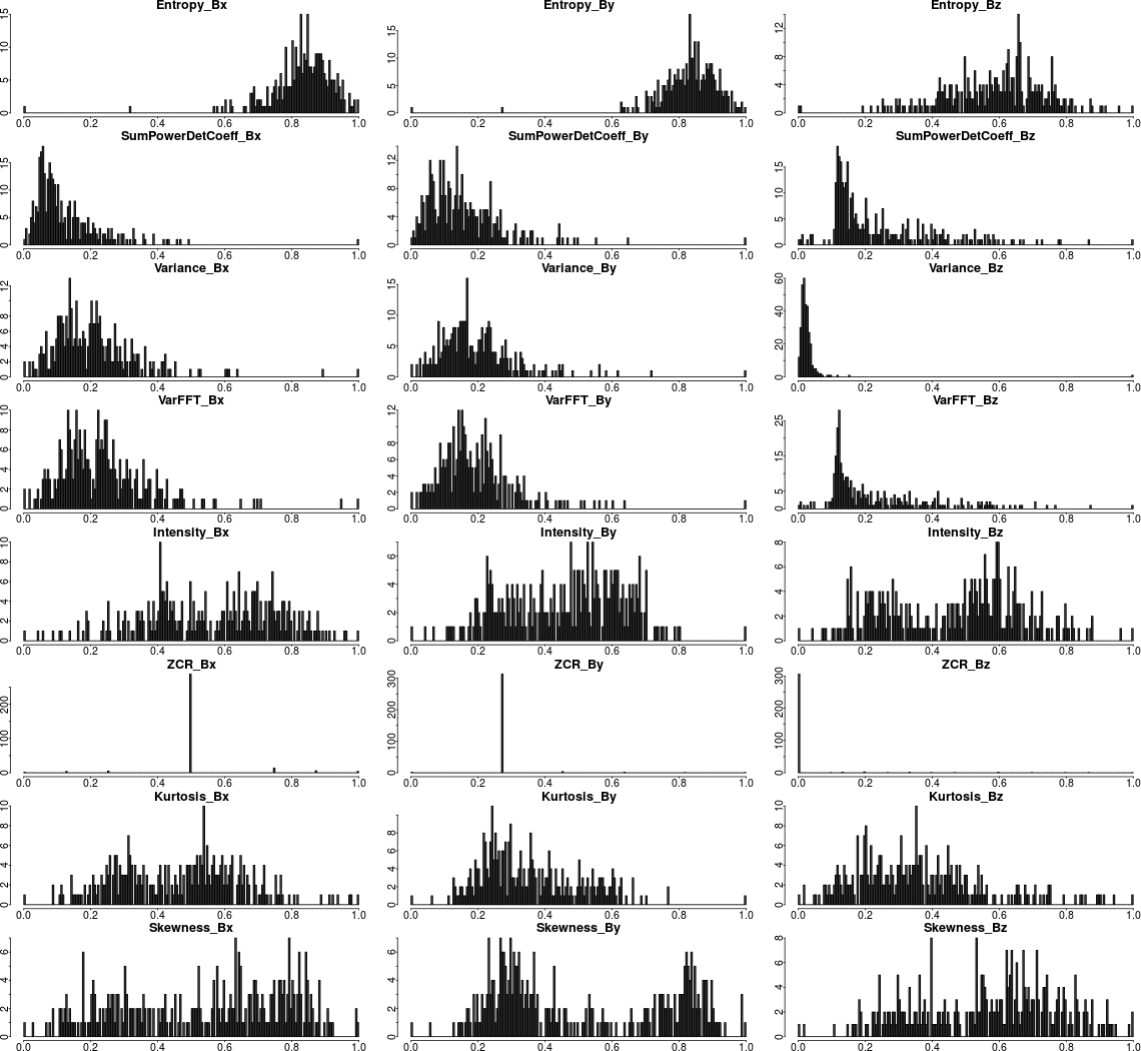
Distribution of features extracted from the data collected in Building A after being pre-processed.

As an initial part of the experimental study, we analyzed the distribution of each variable (feature) in order to observe its behavior (see Figure 5). As a result, it was realized that the distribution of values of some variables had a particular shape, so it seemed reasonable to apply algorithms that work on distributed data, rather than algorithms that rely on distances. This is why we choose the expectation maximization (EM) algorithm [30] to perform the data partition. Nevertheless, we also try another distance-based approach to test its feasibility, in this case, the k-means technique. In particular, the algorithm chosen for k-means was that proposed by Witten *et al.*, [31], which selects the best subset of features based on a lasso-type penalty criterion. The core idea is that clusters may differ in a few variables, so using all of the features would probably produce worse clusters. *A priori*, we do not know which features could lead to optimum partitioning, so it is convenient to use this algorithm, which has been demonstrated to be a better approach than standard k-means and other clustering algorithms, as Witten *et al.* show.

The two algorithms are compared in terms of how they grouped data. As mentioned in the previous subsection, we are interested in analyzing the performance of models using only the three components of the magnetic field and also considering more information, extracted in the form of features. Furthermore, each location $Z_{q}^{\left(t\right)}$ is also used to force the algorithm to group data that are close in space, making the estimation more precise. For this step, the k-means algorithm with feature selection is executed, along with EM, considering the following variables:

1. Bx, By and Bz intensity and magnitude of intensity.
2. All features.
3. All features transformed by principal components analysis (PCA).

After trying different numbers of clusters and configurations of features for each algorithm, the best clustering method and partitioning are to be selected. The results are shown in Table 2. In the case of k-means, the optimum number of clusters chosen is based on the silhouette coefficient, which measures the quality of the clusters, *i.e.*, how well the data have been grouped. This method divides space into only two regions. With respect to the EM algorithm, the criterion for selecting the optimum *k*is the Bayesian information criterion (BIC) [32]. In all cases, except for the configuration with the features Bx intensity, By intensity, Bz intensity and magnitude of intensity, the best partition is also found to be two clusters. If the building were divided into two zones, the estimators would have to predict a huge range of values, so the system would not be scalable or accurate. Moreover, we had identified more than two zones containing possible landmarks (zones where lifts, printers or servers were located), so the expert knowledge would reject these space partitions. However, by using only the magnetic field intensities, the EM algorithm recognizes eight zones with different distributions, all corresponding to those that had been foreseen. As a result, the EM algorithm is used to perform the building space division based on the information provided by the intensity of the three components of the magnetic field and its vectorial magnitude.

Another possibility would be to configure the cluster manually. The underlying idea is that clustering algorithms group data that are similar into the same cluster. Thus, classification is expected to be precise, because it makes distinctions among data that are supposed to be different: the classifier will perform better if the difference between data from different clusters is pronounced. Nevertheless, if data are very similar, the estimator of each zone cannot take advantage of any variance in the same. As a consequence, the estimator would not be as accurate as it could be. This suggests that a trade-off between classification and estimation must be achieved. For this reason, we use the zones detected by the clustering algorithm to rearrange related data into a new configuration of ten zones, instead of eight, that group data that are less similar, but closer in space. The estimation results were expected to be more accurate, and classification worse. Therefore, if a more fine-grained estimation is required, we suggest manually rearranging the clusters resulting from the EM algorithm, although this has the disadvantage that fewer zones can be classified, as mentioned above. Otherwise, if a more general solution is needed, as is the case here, the EM algorithm provides the optimum configuration of zones, although classification is not as accurate.

#### 4.2.4. Landmark Classifier

The landmark classifier assigns each new magnetic field measurement to a specific landmark previously determined by the clustering algorithm. In order to select a suitable classification technique, we analyze the performance of different classifiers. Since using algorithms based on data distribution was proposed previously, we first choose a classifier that works with Gaussian processes, based on the assumption that most features can be modeled by a mixture of Gaussian functions. As we wish to assess the performance of a range of classifier techniques, four totally different classifiers are added so as to cover diverse approaches. The algorithms selected are the following, and their mathematical foundations can be found in the corresponding references:

1. Gaussian process with radial basis function kernel (gaussprRadial) [33].
2. Single C5.0 Tree [34].
3. Soft Independent Modeling of Class Analogy (SIMCA) [35].
4. Multi-layer perceptron with Resilient Backpropagation (Rprop) [36].
5. Bagged Classification And Regression Tree (CART) [37].

**Figure 6:**
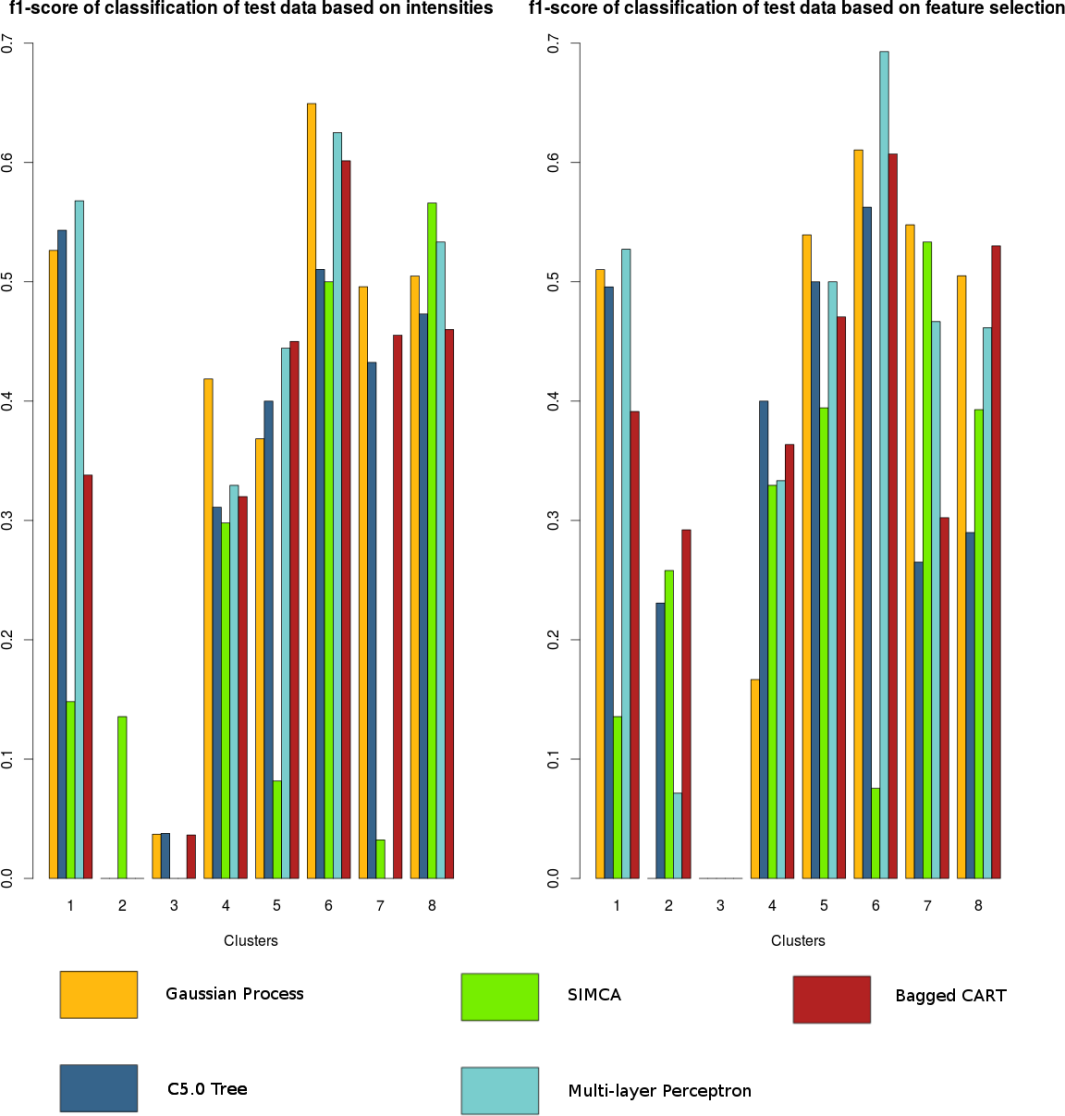
Classification results of automatically-clustered data in Building A.

The results are summarized in Figure 6. We assess the performance with the f1-score metric, whose expression is $F_{1}=2\cdot \frac{\text{precision}\cdot \text{recall}}{\text{precision}+\text{recall}}$, and whose value range is 0 <= *F*_1_ <= 1. Even though it does not consider the true negative rate, it is possible to see whether each class (*i.e.*, zone) has been correctly predicted or not with respect to the other classes, but this is underestimated. As can be seen in Figure 6, the classifier based on Gaussian processes yields more stable results and, unlike the others, is able to predict five zones out of eight with an f1-score higher than 0.5. Furthermore, classification is improved if based on feature selection rather than on intensities alone, unlike in the clustering stage. The variables selected are the following: *Bx, By, Bz intensity; By kurtosis; Bx, By, Bz skewness; Bx, Bz SumPowerDetCoeff; Bz VarFFT*.

If the classification is made in the five zones that *gaussprRadial*computes more accurately, this algorithm would be expected to yield better classification results, as shown in Table 3. Therefore, it seems wise to use this classifier only for these well-predicted zones.

After classifying the zone of the building for each new measurement, we can focus on the magnetic field characterization of the landmark covering this zone and ignore the rest of the space to estimate location.

#### 4.2.5. Location Estimator

Once the magnetic field measurements are correctly classified according to their associated landmark, the zone of the building to which every measurement belongs can be inferred. The next step is to estimate position using the knowledge available for the associated landmark.

In contrast to other works, we propose to estimate each component at a particular location. This system is valid for an estimation made at a fixed height from the floor, so it will yield the (*x, y*) position. This estimated location is referenced to a local coordinate system within the building. Therefore, it is necessary for each particular building to have a specific coordinate system defined. Bearing this in mind, we train different regression algorithms for each zone and coordinate of the 2D position. Our goal is to obtain the optimum estimator at a particular location and component, *i.e.*, *x* and *y*. In particular, we choose two different algorithms, which are described below:

1. Gaussian process with radial basis function kernel: This is the same algorithm that was proposed in the previous section, but in its version that is suitable for regression. We assume that the variables can be modeled by a normal distribution, so it seems reasonable to try with this technique.
2. Bayesian regularized neural network: An artificial neural network consisting of two layers is fitted by making use of regularization to optimize the output function, as described in [38]. This technique improves generalization because, instead of minimizing the mean of the sum of squared errors (MSE), it also takes into account the mean of the sum of squared network weights (MSW). Thus, the objective function becomes $F=\alpha \cdot \text{MSE}+\beta \cdot \text{MSW}=\alpha \cdot \frac{1}{n}{\text{∑}}_{i=1}^{n}{\left(e_{i}\right)}^{2}+\beta \cdot \frac{1}{n}{\text{∑}}_{j=1}^{n}{\left(w_{i}\right)}^{2}$. Estimation of the parameters *α* and *β* is made by Bayesian optimization and the Gauss-Newton algorithm. The trained network tends to be smoother, so it avoids overfitting. The reason for considering this algorithm is that neural networks have been seen to adapt to the particularities of any data, which is why it is widely used in solutions based on soft computing.

At the end of this stage, as many estimators will have been trained as there are magnetic field landmarks (zones) and components (*x, y*), enabling the optimum regression technique to be determined for each zone within the building. As will be shown in the following section, not only is the best estimator for each zone chosen, but also the best features for it. As a consequence of the methodology proposed, each zone will have been attributed the optimum combination of estimator + features to achieve optimum estimation.

The location estimator is the last stage of the offline phase of our proposed location system.

### 4.3. Online Location Phase

**Figure 7:**
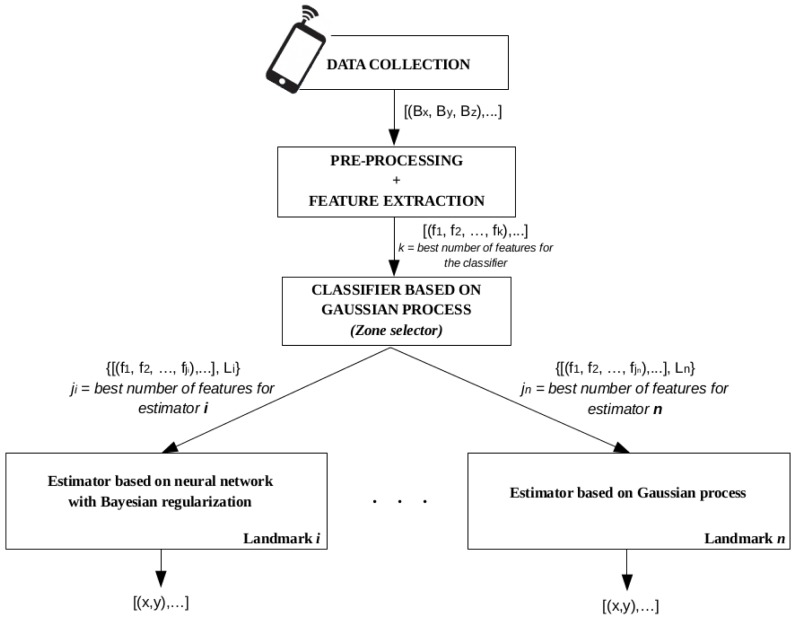
Online phase of the MagicFinger mechanism.

After the offline phase, user location can be estimated using the magnetic field maps generated and the location estimator designed for each zone. A schema of the steps followed during the online phase of the MagicFinger system can be seen in Figure 7. The input data consist of the magnetic field measurements sensed by the user's phone magnetometer. From such measurements, the magnetic field features are extracted in the form of a vector. This feature vector is classified as belonging to a particular landmark cluster. Finally, the user's location is estimated using the corresponding estimator that has been implemented for each landmark.

## 5. Evaluation and Result Analysis

The primary goal of our evaluation process is to verify the practical feasibility of our approach to solve the indoor location problem using the magnetic field vectors sensed by common smartphones. The experimental procedure and results are presented in the following subsections.

### 5.1. Experimental Data Collection

In order to evaluate the MagicFinger mechanism, it is necessary to choose the optimum parameters for the different techniques that make up the mechanism. To achieve this, it is convenient to collect training and test data of the magnetic field distribution throughout the space of interest in the target building. Using the collected data, an optimum configuration for each technique involved can be obtained after analysis of the results associated in terms of location error. Therefore, in this section, we describe the experiments carried out in Building A to obtain the optimum parameters for each technique finally selected, in order to implement the location mechanism proposed, as well as to validate the mechanism with five-times 10-fold cross-validation, or bootstrapping in the case of the estimators, due to the size of the set of samples of each particular zone/cluster.

We have developed an Android application for the HTC One X (S720e) smartphone. This phone is fitted with a Hall-effect geomagnetic sensor3with three axes. The sensor implements a dynamic offset estimation (DOE) algorithm to automatically compensate the magnetic offset fluctuations, thereby making it more resilient to magnetic field variations within the device [39]. In addition, the effect of high frequency ambient noise is mitigated by averaging the measurements prior to sensor calibration. The application is able to gather magnetometer signals at a frequency of 25 Hz and to record them on a database located in an external platform (in the cloud) or in the internal storage space (like an SD card).

After constructing the application for measuring the magnetic field perturbation, we focus on designing the best way to gather data for Building A. Since our intention is to cover the largest possible area, the whole first floor of this building is considered the most suitable place. Because a trade-off between the amount of data and the building size needs to be achieved, we decided to collect data every 150 cm, which results in 323 locations to be measured. At each particular position, an average of 90 readings of the magnetic field are taken by considering all orientations. This is done by placing the smartphone horizontally on a rotating platform, made of plastic, so as to avoid any kind of perturbation, at a height of 1.03 m, which approximately corresponds to the average height at which the user would be looking at their phone. One full measurement takes around 20 s, with samples being taken at every 3∼4 degrees of rotation. Moreover, as we want to consider different types of variability, we perform three data collection campaigns on different days during one week and at different times of the day. In this way, we are able to measure different levels of activity in every zone of the building, meaning that variability in contextual factors is included in our data baseline. Then, using this dataset, the data processing techniques presented in Section 4.2 are analyzed considering different values for their implementation.

The first analysis required in our mechanism concerns the optimum number of clusters to be considered for constructing the classifier, *i.e.*, the optimum number of landmarks in terms of magnetic field distribution. This involves identifying the number of landmarks necessary to achieve a trade-off between the error obtained in the landmark classification and the associated error obtained from the location estimation provided by the estimator for each landmark. The distribution of landmarks identified corresponds to the zones where different electronic and mechanical infrastructure items are sited, such as lifts, printers, servers, laboratories, *etc.*, all of which represent a source of perturbation of the Earth's magnetic field.

The offline training phase of our location system provides a 3D map containing magnetic field features of the building and identifies the best regression technique for each landmark. This map is associated with a predefined position (horizontal) representing the position at which participants would carry their devices.

### 5.2. Analysis of Experimental Results

**Figure 8:**
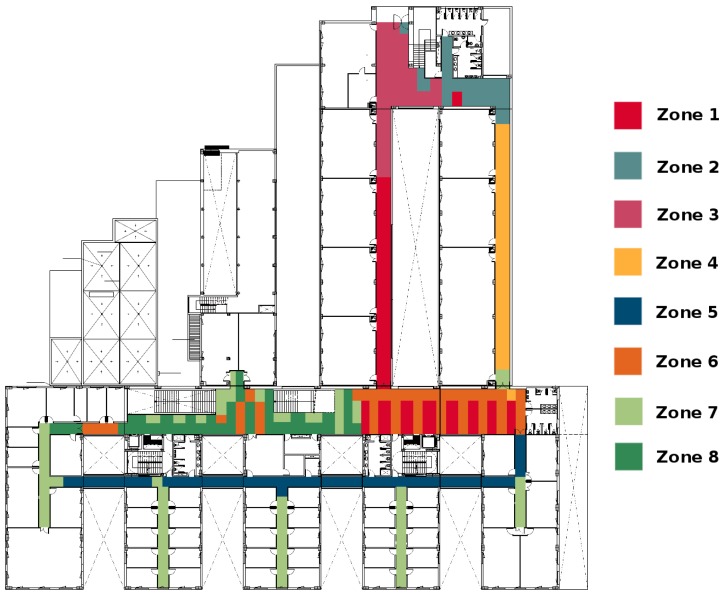
Zones in Building A identified with the EM algorithm using the intensity parameter of the magnetic field.

By considering the map of the building containing the magnetic field profile resulting from the training phase, the classification mechanism, which is responsible for assigning to each new measurement the zone to which it belongs, is evaluated. Firstly, we evaluate the classification success on test data based on clustered training data with an EM algorithm using intensities, which proved to be the best technique, as discussed in the previous section, and using the zones selected manually. These zones are laid out in Figure 8.

**Figure 9:**
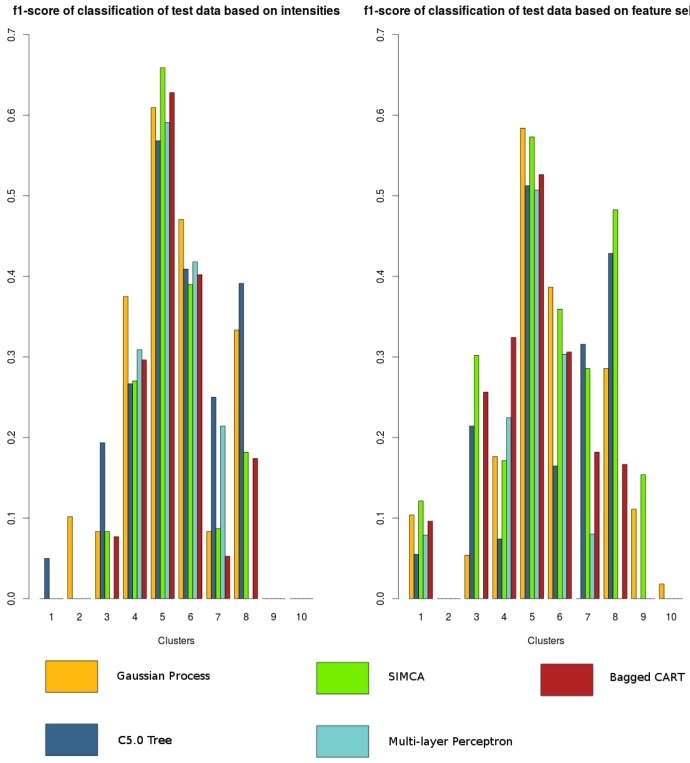
Classification results of manually-clustered data in Building A.

Classification is made using only the magnitude of the magnetic field and the intensities of its three components and performing feature selection on all filtered features. Furthermore, in choosing the best tuning parameters for each algorithm, the package *caret* [40] for R software was found useful. Once the best tuning parameters for each classifier were obtained through cross-validation, they are evaluated using test data that were used for training. As shown in Figure 6 and also described in the previous section, classification on automatically-clustered data can detect up to five zones with considerable precision if the chosen classifier is the one based on Gaussian processes with feature selection. However, this is not the case for manually-clustered data. To confirm this, the same algorithms are tried with our manually-selected zones (see Figure 9). Because our clustered samples are not optimally related (favoring more accurate estimation), the performance of the classifier is clearly worse. Depending on the configuration of technique + features, no more than two or three zones could be predicted when isolated. Therefore, rearranging clusters manually results in a less general solution.

Once we studied the feasibility of classifying both types of zones, we can assess the performance of the estimators. First, we focus on measuring the performance of estimators in the zones detected automatically. For each zone and component (in this case, we predict *x* and *y* positions at a fixed height of 1.03 m above ground level), we train the two algorithms described in Section 4 (Gaussian process and Bayesian regularized neural network). As in the classification phase, we consider measuring precision using only intensities and using the features selected from all of the features. With the aid of the caretpackage, we train the estimators with the best tuned parameters based on training data. After this, we measure the performance of these techniques on test data. The error is measured in terms of RMSE (root mean square error), because it yields the values in the same units as the output of the estimators, *i.e.*, in meters, so the results can be interpreted easily. This measure describes the standard deviation of the residuals, which are the differences between ground truth and predicted data.

Estimation results for the automatically-clustered data are presented in Tables 4 and 5, in which the best technique for each zone and component has been highlighted. It is clear from these results that there is no general algorithm with a combination of features that yields the optimum output, which depends on the type of data for each zone. With our proposed methodology, it is easy to implement the optimum combination of algorithm + features for each zone and component, because after the magnetic field measurement has been classified, it is possible to know in which zone the user is, and the particular estimator is executed for this zone. The estimators selected for each zone of Building A along with their associated features are presented in Table 6.

By analyzing the zones that are best classified in the previous step (namely, Zones 1, 5, 6, 7 and 8), we can see how the error ranges from 6.04 m to 14.25 m in component *x* and from 0.76 m to 10.63 m in component *y*. Although the error obtained seems reasonable, in view of the size of the zones where location is to be solved, it is not suitable for fine-grained estimation of the user's location, but it is suitable for predicting the subzone where the user is. For this reason, we study whether performing an estimation based on manually-clustered data improves these results, as we expect they will. In order to assess this aspect, we train the algorithm that works with Gaussian processes using only the information given by the intensities of the components of the magnetic field. The results are summarized in Table 7.

Based on the results obtained, other algorithms are not assessed, because it is clear that the best results are those obtained when the zones are most naturally heterogeneous and concentrated in space. This is mainly because algorithms obtain more information from data that are not optimally related, while clustering has the opposite effect. However, this has a detrimental effect on classification, which is less precise if data are not automatically clustered. Therefore, fewer zones can be predicted. On the other hand, estimation is less accurate when automatically-clustered data are used. Therefore, it is useful to manually reconfigure the clustered data to obtain a more fine-grained estimation in a few particular zones of the building. Conversely, if we are interested in covering most of the building space, we should cluster data with the EM algorithm and use the zones thus detected. Again, the latter option yields less precise estimation results, but is appropriate for subzone location.

### 5.3. System Validation

Since the purpose of this study is to develop a system as scalable as possible, the proposed methodology is analyzed under different conditions. The goal is to determine whether this methodology can be generalized or needs to be adapted to each particular building. For this reason, the steps followed are the same as those described in the previous section.

The chosen environment is the ground floor of the Psychology Faculty of the University of Murcia (Building B), where we collect data from 151 locations, covering the entire floor. As was done in Building A, measurements are taken every 150 cm. In addition, data are collected taking into account three different situations regarding the activity level of students and workers inside the building and at a height of 1.03 m above the ground level. Test data were gathered two weeks after collecting training data.

**Figure 10:**
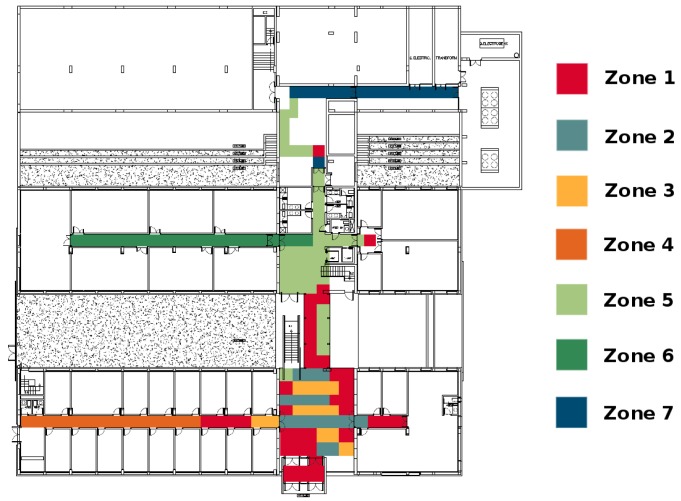
Zones in Building B identified with the EM algorithm using the intensity parameter of the magnetic field.

The next step is to identify zones of the building with similar magnetic field values, using the EM clustering algorithm (based on the intensities of the three components of the magnetic field). Seven distinct zones are identified, confirming that the use of this algorithm yields a fine-grained partitioning of a building. The main advantage is that there are enough clusters to perform an accurate estimation, but not so many zones that the classifier may misclassify them. These zones are depicted in Figure 10.

**Figure 11:**
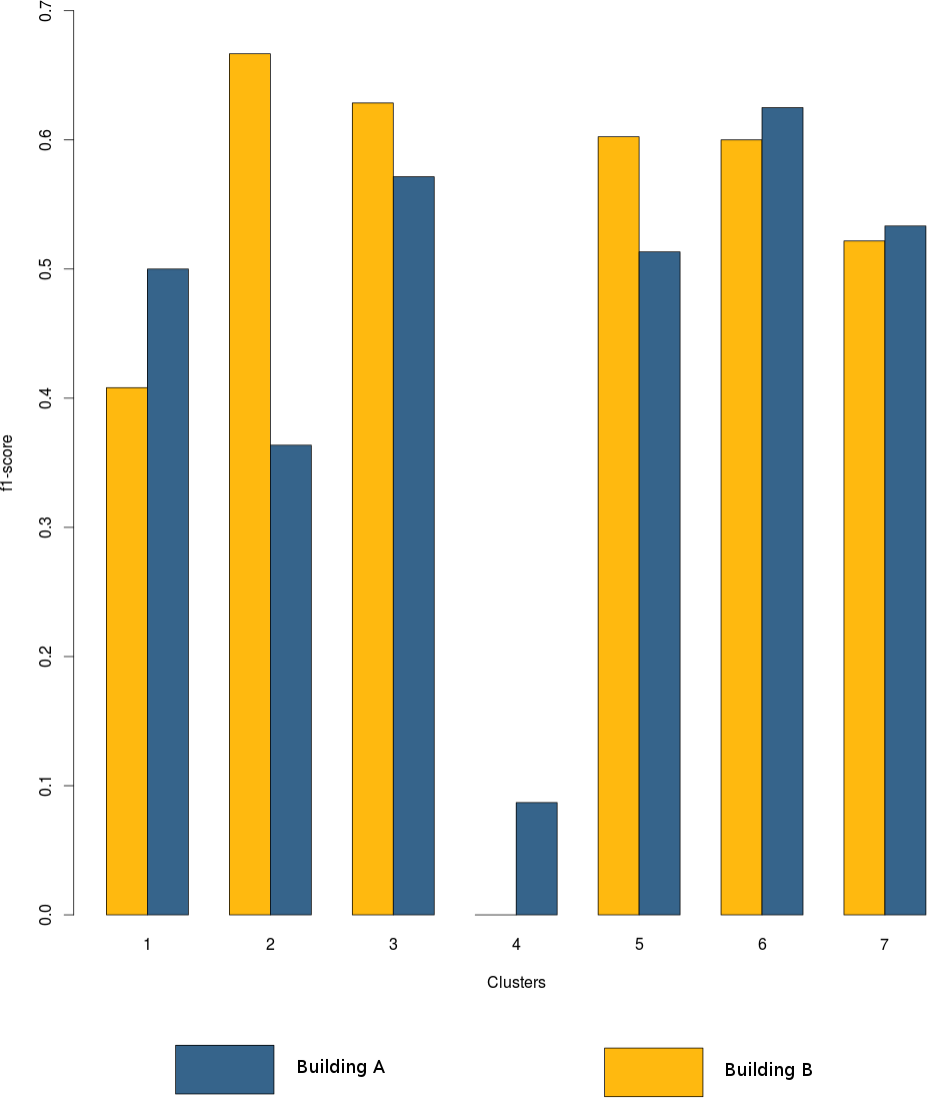
Comparison between classification based on features selected from Building A and B.

Once the data were grouped into zones, the classifier is trained, and the best estimator for each zone is selected. To train the classifier, we choose the Gaussian process, using the variables selected by the Recursive Feature Elimination algorithm. At this point, it is interesting to analyze the performance of this classifier using the features selected for Building A and performing feature selection on data from Building B. As Figure 11 shows and as expected, using the best features for each building yields better results, although there is not a great difference. Furthermore, it is interesting to note that not all zones are correctly predicted, as happened in Building A. Here, classification and estimation is only feasible in Zones 2, 3, 5, 6 and 7. The features selected in Building B are the following: *Bx, By entropy; Bx, By, Bz intensity; Bx, By kurtosis; Bx, By, Bz skewness; Bz sumPowerDetCoeff; Bz VarFFT*. Considering these zones, classification yields the results summarized in Table 8.

The last step of the process is to find the optimum estimator for each well-predicted zone. For this, the two proposed algorithms are analyzed, along with only intensities or with the features selected for each zone. As a result of this process, nine estimators are trained, as shown in Table 9, while Table 10 depicts the accuracies of these predictors. According to these results, most zones have errors of less than 5 m, which allows our system to estimate location fairly accurately. However, some zones lack fine-grained precision, and thus, their estimators are only able to estimate the subzone in which the user is.

The main drawback of MagicFinger is that it is not able to cover the whole building space, although it does cover a significant portion of it. This is due to the fact that there are some zones whose magnetic perturbation cannot be modeled. For these regions, it would be interesting to combine magnetic field data with the information used by the solutions described in Section 2, when performing the classification step. It is important to note that the problem only lies in classification, not in estimation. The reason for this may be that those unclassifiable zones have a considerable difference in perturbation, *i.e.*, perturbation is not as stable there as it is in the other areas, so estimators take advantage of this variation, whereas classifiers suffer the consequences. Estimation results from these particular zones from Building A (Zones 2, 3, and 4) corroborate this fact, as shown in Tables 4 and 5.

As conclusions of the analysis presented here, tackling the problem of indoor location using only the magnetic field and the proposed mechanism is seen to be feasible and to yield reasonably good precision in estimating the user's location, or at least the subzone where the user is located. The results obtained after applying our approach to two different buildings show that, even if the buildings differ in magnetic perturbation and level of activity, the proposed methodology adapts itself to the particular conditions of each building, resulting in a scalable system.

## 6. Conclusions

This paper presents MagicFinger as a novel methodology for solving indoor location based on 3D magnetic field measurements inside buildings obtained from off-the-shelf smartphones. The use of landmarks derived from magnetic field disturbances for the purpose of indoor location has one considerable advantage over radio-based location techniques: no external infrastructure is required. Our experience, presented in this paper, shows that this location approach is a feasible solution for use in indoor environments.

The MagicFinger approach consists of two phases. In an offline phase, the magnetic field profile of the building is mapped, and the optimum parameters are selected for the design of the classifier and estimators responsible for the location. Based on these models, the user position is estimated in an online phase based on magnetic field measurements sensed by the users' phones.

In order to test the feasibility of our approach and to select an optimum set of classifiers and corresponding parameters, a detailed experimental study is performed in two different buildings. This study considers a wide range of computational techniques to assess both the classification success rate and location estimation accuracy. By using magnetic field measurements, MagicFinger provides good robustness and accuracy for buildings with low magnetic field variability. This is possible thanks to the use of all three magnetic field components for location, combined with adequate soft computing techniques. Furthermore, the methodology proposed in this paper partitions a building into several zones, which allows one to identify the best estimator for each zone and, thus, to achieve a more precise estimation of the location.

The experiments, although currently limited to a fixed device height, highlight the potential of MagicFinger to provide a serious low-cost alternative for indoor location. This is borne out by the fact that it is evaluated in two environments with different conditions, yielding similar and promising results for estimating user location or the subzone where the user is located. Therefore, MagicFinger represents a novel methodology for indoor location that optimizes location accuracy, since it is able to adapt to building conditions.

Further work will focus on combining MagicFinger with techniques such as uDirect [41], in order to calibrate magnetic field measurements to a common reference coordinate system. The purpose of this is to make device carrying position irrelevant during the fingerprinting and location estimation phases. In addition, more exhaustive data collection campaigns should be conducted in order to generate a more complete map of the buildings.

## Figures and Tables

**Table 1:** Summary of the features extracted from magnetic field measurements.

	**Feature**	**Description**
1	Entropy	Measurement of the uncertainty associated with the data. Measuring from different positions during a walking activity provides different periodical patterns.
2	SumPowerDetCoeff	Measurement of the power of the coefficients derived from the discrete wavelet data transformation [17].
3	Variance	The variance of the data is considered to be relevant following a similar reasoning to that addressed in [18].
4	VarFTT	The variance of FFT (Fast Fourier Transformation) coefficients between 0.5 Hz and 5.5 Hz, covering the range in which most of the energy involved in daily activities lies [19].
5	Intensity	Analogous to [20], the intensity is calculated as the sum of the derivative of a normalized set of samples.
6	ZCR	Analogous to [21], it measures zero-crossing rate with the zero level set to the signal mean.
7	Kurtosis	Kurtosis measures the peak of the data relative to the normal distribution, as it is suggested in [22–24].
8	Skewness	This is a widely used feature that measures the data symmetry. It is applied, for instance, in [22–24].
9	Correlation coefficient	It represents the ratio of the data covariance. It has been used for activity recognition purposes in [24–27].

**Table 2:** Optimum number of clusters proposed by each clustering algorithm for Building A.

**Algorithm**	**Features**	**Clusters**
k-means with feature selection	Bx variance and Bx VarFFT were selected among all	2
EM	Bx, By, Bz intensity and magnitude of intensity	8
EM	All	2
EM	All transformed by PCA	2

**Table 3:** f1-score of classification using gaussprRadial and 5 zones in Building A.

**Zone**	**f1-Score**
1	0.519
5	0.675
6	0.660
7	0.694
8	0.548

**Table 4:** RMSE (meters) using only intensities based on automatically-clustered data in Building A.

**Zone**	**Surface**	**Bayesian Regularized NN**		**Gaussian Process**	

		***RMSE****_x_*	***RMSE****_y_*	***RMSE****_x_*	***RM*SE***_y_*
1	21 m × 46.5 m	6.31	19.32	6.57	13.09
2	13.5 m × 10.5 m	5.10	3.57	3.91	2.84
3	7.5 m × 18 m	3.17	10.68	2.19	**5.18**
4	1.5 m × 39 m	**0.24**	**5.87**	0.28	7.03
5	63 m × 7.5 m	**14.05**	3.63	17.81	1.17
6	60 m × 4.5 m	14.31	1.73	**12.33**	**1.71**
7	66.5 m × 28.5 m	**14.25**	12.98	18.55	10.91
8	40.5 m × 7.5 m	11.62	1.75	10.59	**1.72**

**Table 5:** RMSE (meters) performing feature selection based on automatically-clustered data in Building A.

**Zone**	**Surface**	**Bayesian Regularized NN**		**Gaussian Process**	

		***RMSE****_x_*	***RMSE****_y_*	***RMSE****_x_*	***RMSE****_y_*
1	21 m × 46.5 m	7.60	26.10	**6.04**	**10.63**
2	13.5 m × 10.5 m	4.07	2.79	**3.65**	**2.60**
3	7.5 m × 18 m	2.02	9.37	**1.73**	5.30
4	1.5 m × 39 m	0.97	5.89	0.71	7.13
5	63 m × 7.5 m	16.22	0.88	17.76	**0.76**
6	60 m × 4.5 m	19.53	1.79	12.75	1.76
7	66.5 m × 28.5 m	19.78	20.64	18.33	**9.06**
8	40.5 m × 7.5 m	10.45	2	**10.05**	1.80

**Table 6:** Estimators selected for each zone in Building A with the features that they take as input.

**Zone**	**Component**	**Estimator**	**Features**
1	x	Gaussian process	Bx, By, Bz entropy & Bx, By, Bz intensity & Bx, By kurtosis & Bx, By skewness & Bx, By, Bz sumPowerDetCoeff & Bx, By, Bz VarFFT& Bx, By, Bz variance & Bx ZCR
	y	Gaussian process	Bx, By entropy & Bx, By, Bz intensity & Bx, By, Bz kurtosis & Bx, By, Bz skewness & Bx, By, Bz sumPowerDetCoeff & Bx, By, Bz VarFFT & Bx, By, Bz variance
5	x	Bayesian regularized NN	Bx, By, Bz intensity & magnitude of intensity
	y	Gaussian process	Bz entropy & Bx, By intensity & Bx kurtosis & By, Bz skewness & By sumPowerDettCoeff & Bx VarFFT & Bx variance
6	x	Gaussian process	Bx, By, Bz intensity & magnitude of intensity
	y	Gaussian process	Bx, By, Bz intensity & magnitude of intensity
7	x	Bayesian regularized NN	Bx, By, Bz intensity & magnitude of intensity
	y	Gaussian process	Bx entropy & Bx, By, Bz intensity & Bx, By kurtosis & By skewness & Bx, By, Bz sumPower-DettCoeff & Bx, By, Bz VarFFT & Bx, By variance
8	x	Gaussian process	By intensity & Bx kurtosis & Bz skewness & By, Bz sumPowerDettCoeff & Bx, By VarFFT & Bx, By variance
	y	Gaussian process	Bx, By, Bz intensity & magnitude of intensity

**Table 7:** RMSE (m) using only intensities based on manually-clustered data in Building A.

**Zone**	**Surface**	**Gaussian**	**Process**

		***RMSE****_x_*	***RMSE****_y_*
1	0 m × 30 m	0	10.15
2	7.5 m × 18 m	2.29	4.91
3	7.5 m × 15 m	2.51	2.31
4	0 m × 31.5 m	0	7.20
5	27 m × 4.5 m	7.34	1.59
6	36 m × 7.5 m	9.11	1.80
7	15.5 m × 13.5 m	4.85	3.05
8	15.5 m × 13.5 m	5.13	4.69
9	15.5 m × 13.5 m	5.18	4.31
10	17 m × 19.5 m	7.16	4.36

**Table 8:** f1-score of classification of 5 zones in Building B.

**Zone**	**f1-Score**
2	0.593
3	0.867
5	0.753
6	0.857
7	0.75

**Table 9:** Estimators for each zone in Building B with the features that they take as input.

**Zone**	**Component**	**Estimator**	**Features**
2	x	Gaussian process	Bx, By, Bz intensity & magnitude of intensity
	y	Gaussian process	Bx, Bz intensity & Bx kurtosis & Bx, By sumPowerDetCoeff & Bx, By VarFFT & Bx, By variance
3	x	Gaussian process	Bx, By, Bz intensity & magnitude of intensity
	y	Bayesian regularized NN	Bx, By, Bz intensity & magnitude of intensity
5	x	Bayesian regularized NN	By entropy & Bx, Bz intensity & Bx kurtosis & Bx, Bz skewness & Bx, By, Bz sumPowerDetCoeff & Bx, By, Bz VarFFT & Bx, By variance & Bz ZCR
	y	Gaussian process	By entropy & Bx, By, Bz intensity & Bx, By, Bz kurtosis & Bx, By skewness & Bx VarFFT & Bx, By variance
6	x	Bayesian regularized NN	Bx, By, Bz intensity & magnitude of B
7	x	Gaussian process	Bx, By, Bz intensity & magnitude of B
	y	Gaussian process	Bx, By, Bz intensity & magnitude of B

**Table 10:** RMSE (m) of estimation in Building B.

**Zone**	**Surface**	**RMSE**	

		***RMSE****_x_*	***RMSE****_y_*
2	9 m×9m	2.45	1.81
3	10.5 m × 7.5 m	2.95	1.54
5	9 m × 34 m	1.44	6.46
6	27 m × 0 m	8.09	0
7	18 m × 9 m	5.04	2.13
